# Phase I pharmacological study of continuous chronomodulated capecitabine treatment

**DOI:** 10.1007/s11095-020-02828-6

**Published:** 2020-05-07

**Authors:** Jeroen Roosendaal, Bart A. W. Jacobs, Dick Pluim, Hilde Rosing, Niels de Vries, Erik van Werkhoven, Bastiaan Nuijen, Jos H. Beijnen, Alwin D. R. Huitema, Jan H. M. Schellens, Serena Marchetti

**Affiliations:** 1 Department of Pharmacy & Pharmacology, The Netherlands Cancer Institute, Louwesweg 6, 1066 EC Amsterdam, The Netherlands; 2 Department of Clinical Pharmacology, The Netherlands Cancer Institute, Plesmanlaan 121, 1066 CX Amsterdam, The Netherlands; 3 Department of Biometrics, The Netherlands Cancer Institute, Plesmanlaan 121, 1066 CX Amsterdam, The Netherlands; 4 Science Faculty, Utrecht Institute for Pharmaceutical Sciences (UIPS), Division of Pharmacoepidemiology & Clinical Pharmacology, Utrecht University, P.O. Box 80082, 3508 TB Utrecht, The Netherlands; 5 Department of Clinical Pharmacy, University Medical Center Utrecht, Heidelberglaan 100, 3584 CX Utrecht, The Netherlands

**Keywords:** capecitabine, chronomodulation, metronomic, phase I

## Abstract

**Purpose:**

Capecitabine is an oral pre-pro-drug of the anti-cancer drug 5-fluorouracil (5-FU). The biological activity of the 5-FU degrading enzyme, dihydropyrimidine dehydrogenase (DPD), and the target enzyme thymidylate synthase (TS), are subject to circadian rhythmicity in healthy volunteers. The aim of this study was to determine the maximum tolerated dose (MTD), dose-limiting toxicity (DLT), safety, pharmacokinetics (PK) and pharmacodynamics (PD) of capecitabine therapy adapted to this circadian rhythm (chronomodulated therapy).

**Methods:**

Patients aged ≥18 years with advanced solid tumours potentially benefitting from capecitabine therapy were enrolled. A classical dose escalation 3 + 3 design was applied. Capecitabine was administered daily without interruptions. The daily dose was divided in morning and evening doses that were administered at 9:00 h and 24:00 h, respectively. The ratio of the morning to the evening dose was 3:5 (morning: evening). PK and PD were examined on treatment days 7 and 8.

**Results:**

A total of 25 patients were enrolled. The MTD of continuous chronomodulated capecitabine therapy was established at 750/1250 mg/m^2^/day, and was generally well tolerated. Circadian rhythmicity in the plasma PK of capecitabine, dFCR, dFUR and 5-FU was not demonstrated. TS activity was induced and DPD activity demonstrated circadian rhythmicity during capecitabine treatment.

**Conclusion:**

The MTD of continuous chronomodulated capecitabine treatment allows for a 20% higher dose intensity compared to the approved regimen (1250 mg/m^2^ bi-daily on day 1–14 of every 21-day cycle). Chronomodulated treatment with capecitabine is promising and could lead to improved tolerability and efficacy of capecitabine.

**Electronic supplementary material:**

The online version of this article (10.1007/s11095-020-02828-6) contains supplementary material, which is available to authorized users.

## Introduction

Capecitabine is an oral pre-pro-drug of 5-fluorouracil (5-FU) and is frequently used for the treatment of colorectal, breast and gastric cancer. After administration, capecitabine is rapidly and completely absorbed and converted into subsequently 5′-deoxy-5-fluorocytidine (dFCR), 5′-deoxy-5-fluorouridine (dFUR) and 5-FU via a three-step enzymatic pathway involving carboxyl esterase, cytidine deaminase and thymidine phosphorylase (TP), respectively [1, 2]. Approximately 80% of 5-FU is catabolized to inactive metabolites. A small proportion of 5-FU is intracellularly anabolized to the cytotoxic metabolites 5-fluorouridine 5′-triphosphate (FUTP), 5-fluoro-2′-deoxyuridine 5′-triphosphate (FdUTP), and 5-fluoro-2′-deoxyuridine 5′-monophosphate (FdUMP) [3, 4]. The main mechanism of action is inhibition of the enzyme thymidylate synthase (TS), which is essential for DNA synthesis [5, 6]. Dihydropyrimidine dehydrogenase (DPD) is the enzyme that catalyzes 5-FU degradation into dihydro-5-FU. Dihydro-5-FU is eventually converted to fluoro-β-alanine (FBAL), which is cleared renally [1, 7].

The recommended dose (RD) of capecitabine is 1250 mg/m^2^ twice daily (BID) on day 1–14 of a 21-day cycle [8]. In early clinical phase I studies, both continuous and intermittent dosing regimens were examined [9, 10]. For continuous capecitabine treatment, the RD was 666 mg/m^2^ BID [10], which is a 20% lower dose intensity than for intermittent treatment [9]. Intermittent and continuous treatment schedules have been compared in a phase II clinical trial and showed similar efficacy [11]. Although the intermittent dosing regimen was recommended for further clinical evaluation, diarrhoea, hand-foot syndrome, vomiting, nausea and stomatitis were more frequently reported with the intermittent schedule than the continuous capecitabine treatment [11].

The time of dose administration could also influence tolerability of capecitabine. In previous studies, 5-FU metabolism demonstrated circadian rhythmicity [12–14]. Chronomodulated and constant-rate infusion with intravenous 5-FU have been compared in a randomized trial [15]. Chronomodulation was achieved by nocturnal administration of 5-FU, since peak activity of DPD and trough TS activity were expected during the night. The 5-FU chronomodulated schedule was more effective and less toxic than constant-rate infusion of 5-FU [15].

Recently, we examined the circadian rhythmicity in DPD and TS activity in healthy volunteers [16]. At 2.00 a.m. a peak in DPD activity (which was about 50% higher compared with afternoon activity) and trough TS activity were reported [16]. Adaptation to the circadian rhythm of DPD may result in a more constant 5-FU exposure. This might benefit patients, as exposure to 5-FU at trough TS activity has been associated with improved 5-FU safety and tolerability [17]. Based on these data, we hypothesized that chronomodulated capecitabine therapy would improve treatment tolerability by administering the highest capecitabine dose at night during maximum DPD activity and trough TS activity. Since continuous BID capecitabine treatment was better tolerated than intermittent therapy [11], chronomodulation was expected to result in even better tolerability, which potentially could lead to increased dose intensity.

The aim of this phase I study was to determine the maximum tolerated dose (MTD), dose-limiting toxicity (DLT), pharmacokinetics (PK) and pharmacodynamics (PD) of continuous chronomodulated BID capecitabine therapy.

## Methods

### Patient selection

Patients aged ≥18 years, with advanced solid tumours potentially benefiting from capecitabine treatment and adequate bone marrow, hepatic and renal function were eligible for enrolment. Patients with known DPD deficiency caused by genetic polymorphisms in *DPYD* (DPYD*2A or c.2846A > T) were excluded.

### Study design

This was a phase I, open label, dose-escalation study. Patients received capecitabine tablets (150 mg and 500 mg) on day 1–21 of a 21-day cycle until disease progression, unacceptable toxicity, or patient refusal. Capecitabine was administered with water within 30 min after a light meal, both in the morning and late evening. A classical 3 + 3 dose escalation design was applied. [18] The capecitabine dose was escalated according to five predefined dose levels (1000, 1275, 1600, 2000 and 2550 mg/m^2^ total daily dose). The total daily dose was divided in morning and evening doses that were administered at 9:00 h and 24:00 h (± 1 h), respectively, according to a 3:5 (morning: evening) ratio, based on the 3:5 ratio in trough to peak DPD activity observed in healthy volunteers [16]. The MTD was expanded to a maximum of 12 patients. DLT period was defined as the first three weeks of treatment. Toxicity was assessed weekly during the first treatment cycle and at the end of each subsequent cycle according to the Common Terminology Criteria for Adverse Events (CTC-AE) version 4.03. Tumour response was evaluated every two treatment cycles according to the Response Evaluation Criteria in Solid Tumors (RECIST) version 1.1 [19]. The study protocol was approved by the local ethical committee and was performed in compliance with Good Clinical Practice guidelines and the WHO Declaration of Helsinki. The study was registered in the Dutch Trial Registry (http://www.trialregister.nl, study identifier: NTR4639).

### Pharmacokinetic analyses

In order to examine circadian variability, the plasma PK of capecitabine, dFCR, dFUR, 5-FU, and FBAL were examined during day- and nighttime. Peripheral blood was collected at pre-dose and 0.5, 1, 1.5, 2, 3, 5, 11, 15 h after capecitabine intake at 9:00 h on day 7 of treatment and during the following night (day 8), 0.5, 1, 1.5, 2, 3, 5 and 9 h after capecitabine intake at 24:00 h. Blood samples were collected in lithium-heparinized tubes, which were centrifuged for 10 min at 1500 *g* and 4°C after collection. Isolated plasma was stored at −70°C until further analysis.

As an exploratory objective, the intracellular PK of FUTP, FdUTP and FdUMP in peripheral blood mononuclear cells (PBMCs) were determined at pre-dose, 1.5 and 3 h after capecitabine intake at 9:00 h on day 7 of treatment. For this, PBMCs were isolated from peripheral heparinized blood using Ficoll-paque density gradient and counted using previously described procedures [20]. Capecitabine and metabolite concentrations were quantified using validated liquid chromatography coupled to tandem mass spectrometric (LC-MS/MS) methods [20, 21].

Non-compartmental plasma PK analyses (NCA) were performed using a validated script in R version 3.3.0 [22]. The following individual PK parameters were extracted: the maximum plasma concentrations (*C*_*max*_), the time to reach maximum plasma concentration (*t*_*max*_), and the area under the plasma concentration-time curve up to 5 h post-dose (AUC_0-5h_) for capecitabine, dFCR, dFUR and 5-FU, and the AUC extrapolated to infinity (AUC_0-inf_) for FBAL. Paired *t*-tests were performed for statistical comparison of the AUC_0-5h_ after morning and evening administration of capecitabine.

### Pharmacodynamic analyses

Circadian variability in DPD and TS activity were examined. DPD and TS activity in PBMCs (DPDA_pbmc_ and TSA_pbmc_) were determined at several time points during the day: at pre-dose, 1.5, 11 and 15 h after capecitabine intake at 9:00 h on day 7 and 1.5 h after capecitabine intake at 24:00 h (day 8). In addition, DPDA_pbmc_ and TSA_pbmc_ were determined at screening (within 3 days before treatment). PBMCs were isolated from peripheral heparinized blood using Ficoll-Paque density gradient centrifugation and stored at −80°C until further analysis. DPDA_pbmc_ and TSA_pbmc_ were determined using validated radio-assays [23–25].

The applicability of the dihydrouracil to uracil (DHU:U) ratio in plasma, as a marker for DPD activity, was explored using the same plasma samples as for PK analysis. Uracil and dihydrouracil levels were quantified using a validated LC-MS/MS method [26], after which DHU:U molar ratios were calculated.

To explore the treatment effect of capecitabine on the TP phenotype, TP activity in PBMCs (TPA_pbmc_) was determined at screening, day 7 at pre-dose (9:00 h), and end of treatment using a previously developed assay. [27]

Variability in DPDA_pbmc_ and TSA_pbmc_ was examined using repeated measures analysis of variance (rANOVA) and the nonparametric Friedman test, respectively. The difference between TPA_pbmc_ at screening and day 7 was examined by the paired *t*-test. Statistical difference was considered significant at *p*-values <0.05.

## Results

In total, 25 patients were enrolled in the study between July 2014 and February 2019, of which 22 patients were evaluable for safety. Patient characteristics are summarized in Table I. The median (range) number of administered treatment cycles was 4 (1–11).

**Table I:** Demographic and Disease Characteristics

Characteristic	Number of patients	%
Total number of patients	25	100
Gender		
Male	11	44
Female	14	56
Ethnic origin		
Caucasian	24	96
Creole	1	4
Age		
Median (range), years	64 (40–78)	
WHO performance status		
0	12	48
1	12	48
2	1	4
Primary tumor type		
Colorectal	10	40
SCLC	2	8
Head and neck	2	8
Other	11	44
Stage of cancer		
Locally advanced	1	5
Metastatic	24	95
Prior treatment		
Chemotherapy	24	96
Radiotherapy	13	52
Surgery	16	64
Immunotherapy	12	48

Abbreviations: WHO, world health organisation; SCLC, small cell lung cancer

### Treatment tolerability

Overall, continuous chronomodulated capecitabine therapy was well tolerated. The most commonly reported toxicities were fatigue (68%), hand-foot syndrome (55%), nausea (45%), and diarrhoea (36%). An overview of the observed adverse events possibly, probably or definitely related to study treatment is summarized in Table II.

A total of six serious adverse events (SAEs) were reported, of which one (i.e., grade 3 diarrhoea) was possibly related to study treatment. Other SAEs were grade 3 Ileus (3x), grade 3 urinary tract infection (1x), and grade 4 haemorrhage (1x).

A total of 14 dose reductions during treatment were reported in 10 patients, caused by, hand-foot syndrome (13x), diarrhoea (1x), and anaemia (1x). Median time to first dose reduction was 30 days (range 17–70). Dose delays occurred in 4 patients, due to hand-foot syndrome (4x) and neutropenia (1x).

### Dose-limiting toxicity and maximum tolerated dose

Table III gives an overview of the examined dose levels and experienced DLTs. Three DLTs were observed in two patients at the highest dose level (grade 3 hand-foot syndrome (2x) and grade 3 diarrhoea (1x)). The MTD was established at 2000 mg/m^2^/day, with no experienced DLTs at this dose level.

**Table II:** Treatment-Related Adverse Events in all Cycles by Dose Level

	**Dose level 1**		**Dose level 2**		**Dose level 3**		**Dose level 4**		**Dose level 5**		**Total**	
**Number of patients**	*n* = 3		*n* = 3		*n* = 3		*n* = 8		*n* = 5		*n* = 22	
**CTCAE grade toxicity**	Gr. 1–2	Gr. 3	Gr. 1–2	Gr. 3	Gr. 1–2	Gr. 3	Gr. 1–2	Gr. 3	Gr. 1–2	Gr. 3	N	%
**Toxicity**												
Fatigue	1		2		3		6		2	1	15	68
Hand-foot syndrome			1		1		5		2	3	12	55
Nausea	1		1		3		3		2		10	45
Diarrhoea	2					1	1		2	2	8	36
Anorexia	1		1		1		2		1		6	27
Peripheral sensory neuropathy			1				3		1		5	23
Anemia							1	2	1		4	18
Vomiting					2		1		1		4	18
Blood bilirubin increased	1				1		1				3	14
Dry skin							2		1		3	14
Dysgeusia					1		1		1		3	14
Dry mouth							1		1		2	9
Oral mucositis							1		1		2	9
Nail discoloration									2		2	9
Neutropenia							1		1		2	9
Rash					1				1		2	9

Treatment-related adverse events observed in ≥5% of patients treated with chronomodulated capecitabine or ≥ grade 3. Abbreviations: CTCAE, Common Terminology Criteria for Adverse Events; Gr., grade; *n*, number of subjects

### Pharmacokinetics

**Fig. 1:**
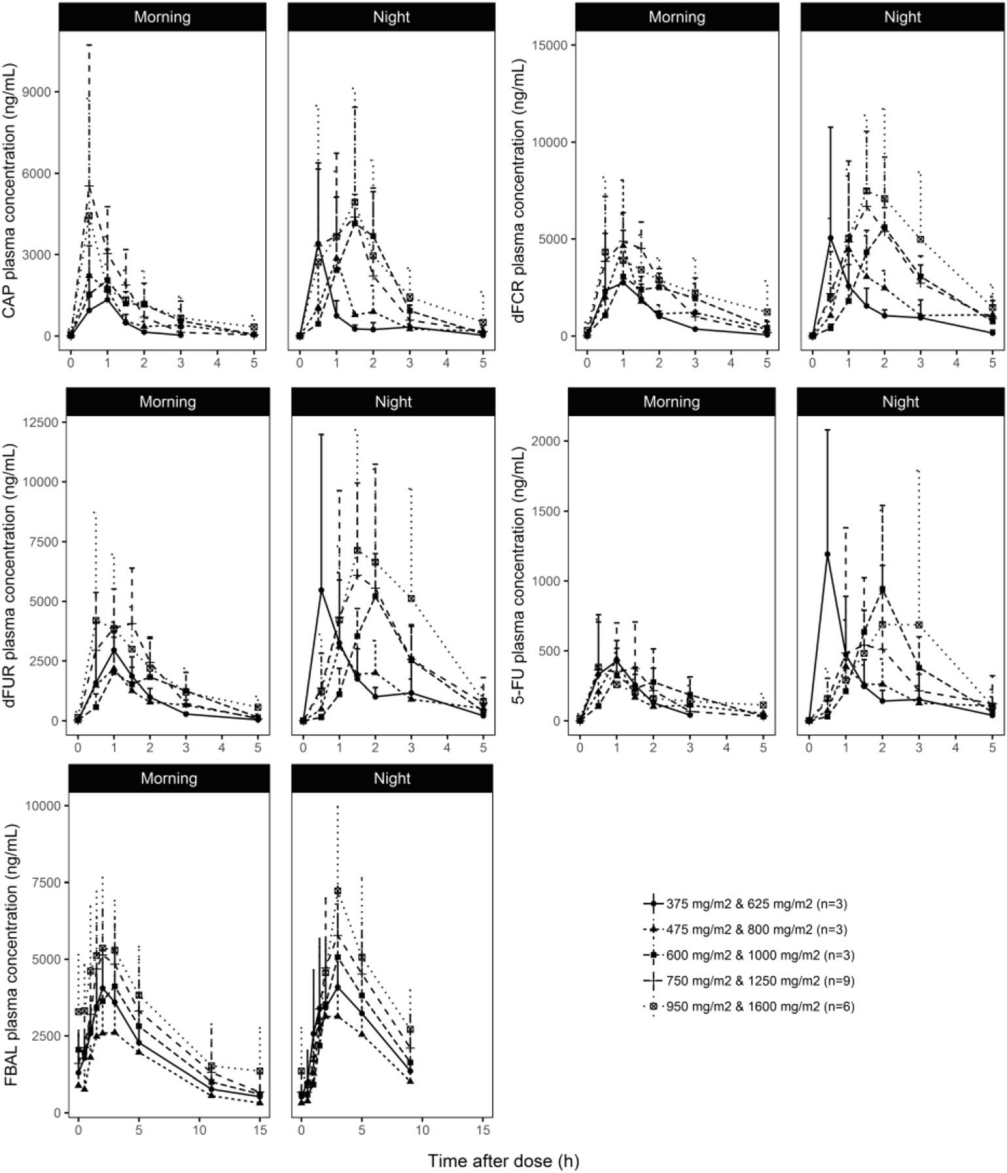
Mean (+SD) plasma concentration-time profiles of capecitabine (CAP), 5′-deoxy-5-fluorocytidine (dFCR), 5′-deoxy-5-fluorouridine (dFUR), 5-fluorouracil (5-FU) and fluoro-β-alanine (FBAL) after dose administration in the morning (at 9:00 h) and at night (24:00 h) for dose level 1–5 on treatment day 7 and 8, respectively. (n = 24).

**Fig. 2:**
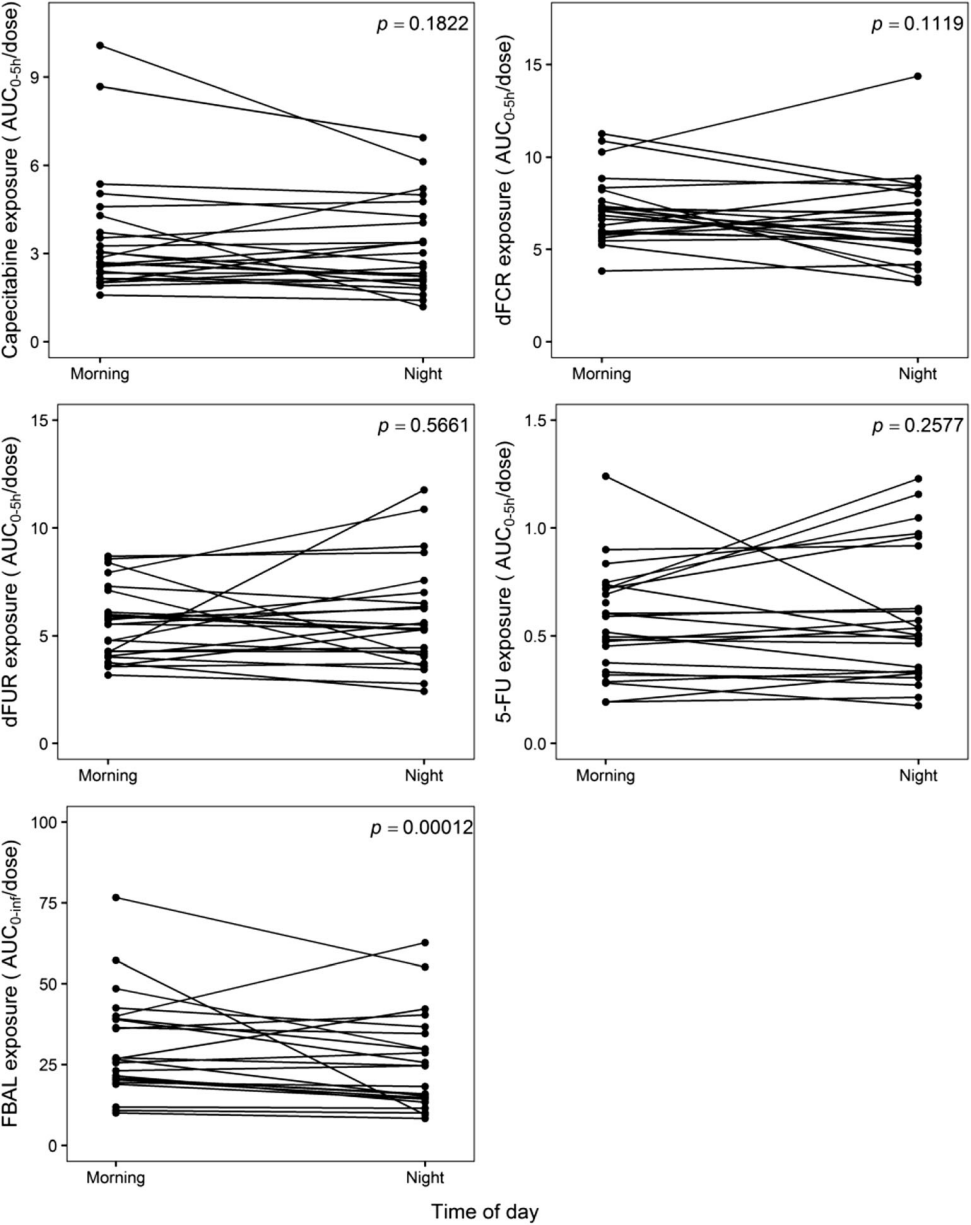
Dose-normalized area under the plasma concentration-time curve up to 5 h (AUC_0-5h_, μg*h/mL) for capecitabine (CAP), 5′-deoxy-5-fluorocytidine (dFCR), 5′-deoxy-5-fluorouridine (dFUR), 5-fluorouracil (5-FU) and extrapolated from zero to infinity (AUC_0-inf_) for fluoro-β-alanine (FBAL) after dose administration in the morning (at 9:00 h) and in night (24:00 h) on treatment day 7 and 8, respectively (*n* = 24).

The mean plasma concentration-time profiles for capecitabine, dFCR, dFUR, 5-FU, and FBAL are shown per dose level and time of day in Fig. 1. Results of the NCA are summarized in Supplementary Table 1. The dose-normalized AUC_0-5h_ are shown in Fig. 2. As shown in this figure, dose-normalized exposure to capecitabine, dFCR, dFUR and 5-FU were not statistically different between daytime and nighttime. For FBAL, daytime exposure was significantly higher than at night (*p* = 0.00012).

**Fig. 3:**
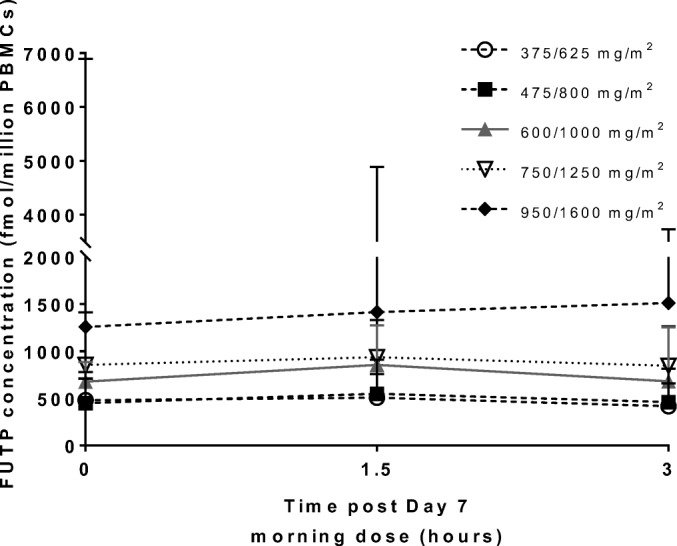
Intracellular peripheral blood mononuclear cell concentrations of 5-fluorouridine-5′-triphosphate (FUTP) after 7 days of chronomodulated capecitabine treatment at indicated morning/evening doses (mean + SD).

Results on the intracellular PK at day 7 are shown in Fig. 3. For all patients, only FUTP was detectable in PBMCs. FUTP concentrations increased per dose level, with a 4.1-fold increase in dose level 5 as compared to dose level 1.

**Table III:** Overview of Dose Levels and Dose-Limiting Toxicities (DLTs)*

**Dose level**	**Capecitabine (mg/m**^**2**^**)** **at 9:00 h / 24:00 h**	**Number of patients**	**Patients experiencing DLT**
1	375 / 625	3	None
2	475 / 800	3	None
3	600 / 1000	3	None
4	750 / 1250	8	None
5	950 / 1600	5	2

*DLT was defined as any of the following events occurring in the first 3 weeks of treatment considered to be at least possibly, probably or definitely related to study treatment: ≥ grade 3 non-hematologic toxicity (other than alopecia, inadequately treated nausea, vomiting or diarrhoea), thrombocytopenia grade 4 or grade 3 associated with bleeding events, grade 4 neutropenia, grade 3 febrile neutropenia, ≥ grade 3 anaemia, and/or a dose interruption of more than 7 days due to toxicity

*§ A total of 22 patients out of the 25 enrolled were evaluable for safety. One patient in dose level 5 was not evaluable for DLT, due intake of an incorrect capecitabine dose during the first 7 days of treatment. Two patients only received one treatment cycle due to symptomatic deterioration related to quick disease progression*

### Pharmacodynamics

**Fig. 4:**
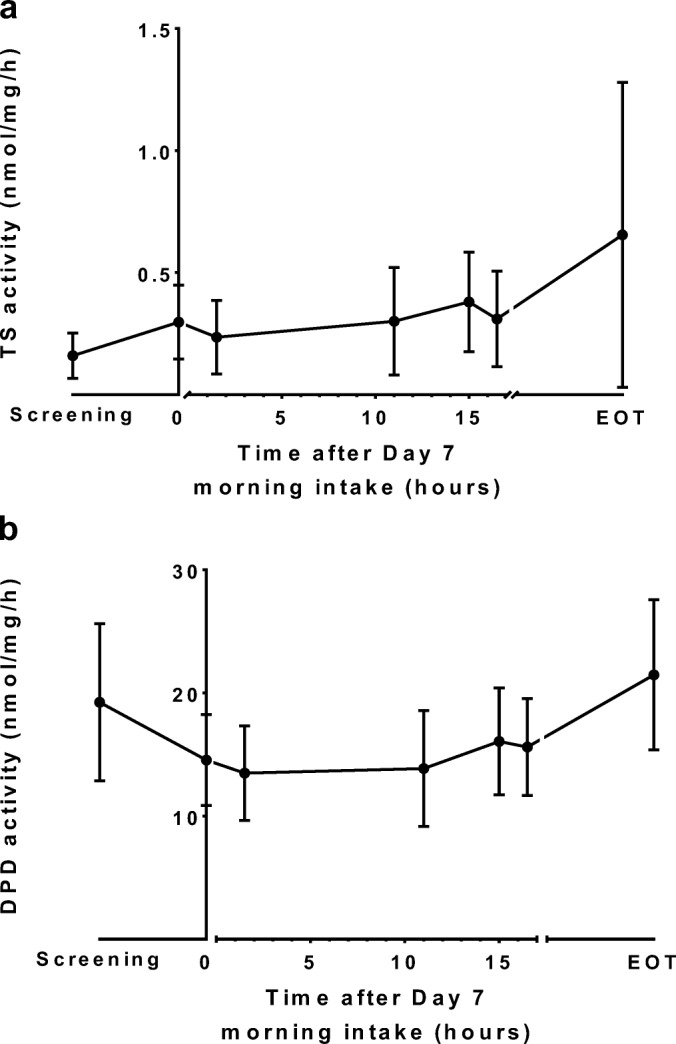
**(a)** Thymidylate synthase (TS) and **(b)** dihydropyrimidine dehydrogenase (DPD) activity (mean ± SD) in peripheral blood mononuclear at screening, day 7/day 8, and end of treatment (EOT) in patients receiving chronomodulated capecitabine treatment (n = 24). Capecitabine was administered at t = 0 h and t = 15 h.

TSA_pbmc_ is shown in Fig. 4a. The median (range) TSA_pbmc_ at screening was 0.134 (0.035–0.584) nmol/mg/h and was significantly induced to 0.301 (0.030–0.677) nmol/mg/h on day 7 at 9:00 h (*p* < 0.0001). TSA_pbmc_ declined again 1.5 h after the dose administration on day 7 (*p* < 0.001). Maximum TSA_pbmc_ was measured at 15 h post-dose (24:00 h) with median (range) activity of 0.346 (0.101–0.729) nmol/mg/h, which was significantly higher than the observed TSA_pbmc_ 1.5 h after dose administration at 9:00 h (*p* < 0.0001).

DPDA_pbmc_ is shown in Fig. 4b. The mean (±SD) DPDA_pbmc_ at screening was 19.2 (± 6.4) nmol/mg/h, which was significantly higher than day 7 at 9:00 h (*P* < 0.001). Significant intra-day variabilities in DPDA_pbmc_ trough and peak activity were observed at 10:30 h and 24:00 h (p < 0.0001), and were 13.5 (±3.8) and 16.1 (±4.3) nmol/mg/h, respectively.

**Fig. 5:**
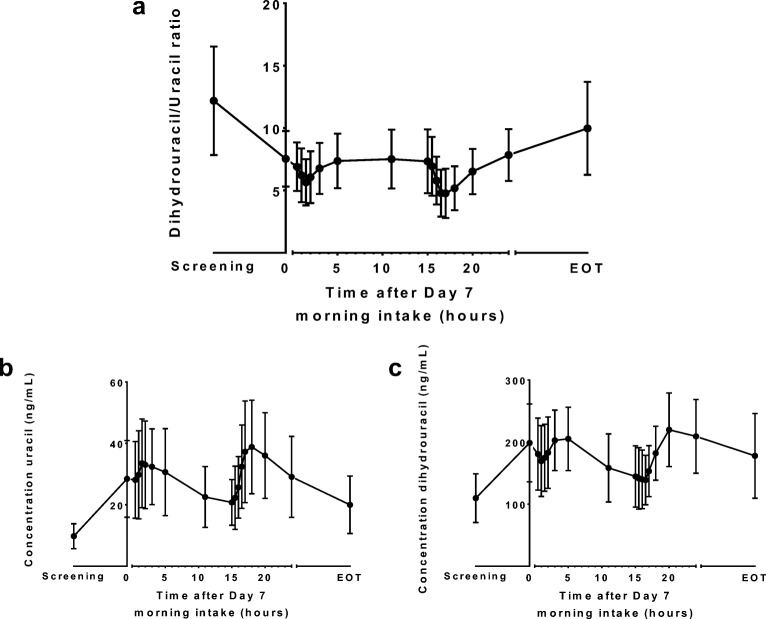
**(a)** The dihydrouracil:uracil plasma ratio, and **(b)** uracil and **(c)** dihydrouracil plasma levels at screening, day 7/day 8, and end of treatment (EOT) in patients receiving chronomodulated capecitabine treatment (mean ± SD, n = 24). Capecitabine was administered at t = 0 h and t = 15 h.

The DHU:U ratio (mean ± SD) in plasma decreased from 12.2 (± 4.4) at screening to 7.5 (± 2.2) on day 7 at 09:00 h (Fig. 5). Pronounced intra-day variability was observed, with mean DHU:U ratio trough levels at 10:30 h and 02:00 h.

There was moderate between-subject variability in TPA_pbmc_ at screening with a mean (±SD) value of 2676 (±1260) nmol/mg/h. There was no significant change in TPA_pbmc_ observed during treatment (Supplementary Fig. 1).

### Tumour response evaluation

Three partial remissions (12%) in patients with breast, ovarian, and neuroendocrine cancer, respectively, were reported. No complete remissions were observed. Thirteen patients (52%) had stable disease as best response. Eight patients (33%) had progressive disease at the first response evaluation.

## Discussion

The present phase I study evaluated MTD, DLT, safety, PK and PD of continuous chronomodulated capecitabine therapy. The total capecitabine dose was stepwise increased from 1000 mg/m^2^/day up to 2550 mg/m^2^/day. The MTD was established at 2000 mg/m^2^/day (750/1250 mg/m^2^/day), with major observed adverse events being grade 1–2 hand-foot syndrome and fatigue. The safety profile overall and at the MTD was in line with previous phase I-III studies of continuous and intermittent capecitabine therapy [9, 10, 28]. The established MTD of 2000 mg/m^2^/day exceeds the previously determined recommended daily dose for regular treatment with continuous capecitabine of 1331 mg/m^2^/day (666 mg/m^2^ BID) [10]. Furthermore, the dose intensity at the MTD was 20% higher than the dose intensity of the currently approved regimen (1250 mg/m^2^ BID on day 1–14 of every 21-day cycle), and even 50% higher than the dose intensity of the intermittent regimen most often used in clinical practice (1000 mg/m^2^ on day 1–14 of every 21-day cycle).

Several chronomodulated treatment strategies for capecitabine have been evaluated in phase II studies [29–33]. In these studies, the total daily capecitabine dose was divided in two or three dosing moments with highest capecitabine dose administered between 18:00–20:00 h [30, 31], at 23:00 h [29, 32], or at 24:00 h [33]. In these studies, chronomodulated capecitabine was combined with oxaliplatin [29–33], and radiotherapy [33]. The examined chronomodulated capecitabine regimens were well tolerated, except in the study performed by Qvortrup et al. [30]. They did not find improved treatment tolerability of chronomodulated capecitabine in combination with oxaliplatin compared to standard capecitabine plus oxaliplatin [30]. The reason for this could be that 80% of the capecitabine daily dose was administered between 18:00 and 20:00 h. According to our finding [16], high-dose capecitabine administration between 18:00 and 20:00 h could be too early to achieve adequate chronomodulation. Indeed, at that time of day, DPD activity is around the baseline value. Due to rapid elimination [2], most of 5-FU is probably degraded before DPD peak activity is encountered. A phase I study of intermittent capecitabine chronotherapy, in which 25% of daily dose was administered at 8:00 h, 25% at 18:00 h and 50% at 23:00 h, on day 1–14 of each 21-day cycle, demonstrated good treatment tolerability [34]. At the declared MTD level of 2750 mg capecitabine per day, only one out of nine patients experienced DLT. Our current findings are in line with previously reported results on chronomodulated capecitabine therapy.

In our study circadian rhythmicity in the dose normalized plasma exposure of capecitabine, dFCR, dFUR and 5-FU was not observed. FBAL exposure was significantly higher during daytime. This may be due to relatively high FBAL concentrations already at pre-dose (9:00 h). Most likely, this finding does not have any clinical implications, since FBAL is an inactive metabolite.

In all treated patients, only FUTP concentrations were quantifiable in PBMCs, with no concentrations above the limit of detection for FdUTP and FdUMP. This is in line with previous research, where only FUTP could be quantified after capecitabine therapy. [35] Interestingly, intracellular FUTP concentrations increase per dose level, with highest levels observed in two patients experiencing DLT.

In healthy volunteers, we previously found that DPD activity in PBMCs demonstrated pronounced circadian rhythmicity, while in the liver, this effect was only minor, as measured by the DHU:U ratio in plasma [16]. Circadian rhythmicity might be regulated in a tissue-specific manner [36]. It could be that DPD activity in liver tissue is not subject to noticeable circadian rhythmicity, explaining the absence of circadian rhythmicity in 5-FU plasma exposure. On the other hand, peripheral 5-FU metabolism could be regulated in a circadian manner, which could contribute to improved treatment tolerability.

TSA_pbmc_ demonstrated peak activity at night, opposite to the trough activity at night observed in healthy volunteers [16]. TSA_pbmc_ was partly inhibited 1.5 h after each dose of capecitabine. This temporary reduction in TSA_pbmc_ is most likely a direct consequence of target inhibition by the intracellularly activated metabolite FdUMP. Previous research demonstrated that TS protein expression is subject to auto-regulation by binding to its own mRNA, preventing protein translation. Binding of FdUMP to TS inhibits this mRNA binding and results in an increase of TS protein levels [37]. As a consequence, capecitabine therapy could lead to TSA_pbmc_ upregulation and a disturbance of the circadian rhythm, as observed in this trial.

Although less pronounced than in healthy volunteers, DPDA_pbmc_ displayed a circadian rhythm during capecitabine treatment. This finding supports our rationale for capecitabine chronotherapy. The less pronounced DPDA_pbmc_ rhythmicity as compared to healthy volunteers may may be caused by capecitabine treatment, or by other factors such as disease status [38]. As for TSA_pbmc_, a partial inhibition of DPDA_pbmc_ was observed 1.5 h after each capecitabine dose. This temporary decrease may be caused by competition between intracellular 5-FU and the ^3^H-Thymine substrate used for the DPDA_pbmc_ assay.

Both the DPDA_pbmc_ and the DHU:U plasma ratio decreased between screening and day 7 of therapy. In addition, a temporary decrease in the DHU:U plasma ratio was observed following capecitabine administration, inversely related to 5-FU exposure in plasma. This last finding is most likely explained by competition between endogenous uracil and 5-FU for DPD conversion. This potential treatment effect should be taken into account when the DPD phenotype is explored during capecitabine treatment.

## Conclusion

Chronomodulation represents a promising strategy as it could lead to improved tolerability and efficacy of capecitabine through achievement of an increased dose intensity in comparison with the currently approved dose regimen. Although questions on PK/PD relationships remain, the results observed in terms of observed MTD by implementation of a chronomodulated capecitabine treatment are encouraging. Additional research is required to evaluate treatment efficacy using the continuous chronomodulated treatment regimen. If further confirmed, this concept could lead to development of a delayed release formulation of capecitabine to allow for nocturnal peak exposure to 5-FU in a patient-friendly way.

## Electronic supplementary material

ESM 1 (PDF 612 kb)
