# Supplementary material for: Phase I pharmacological study of continuous chronomodulated capecitabine treatment
Source: Pharm Res. 2020 May 7;37(5):89. doi: 10.1007/s11095-020-02828-6 (PMC7205843; doi:10.1007/s11095-020-02828-6)

Supplementary Table 1. Pharmacokinetic parameters for capecitabine (CAP), 5'-deoxy-5-fluorocytidine (dFCR), 5'-deoxy-5-fluorouridine (dFUR), 5-fluorouracil (5-FU) and fluoro-β-alanine (FBAL), grouped per dose level and time of capecitabine administration.

		Dose level 1		Dose level 2		Dose level 3		Dose level 4		Dose level 5	
**Number of subjects**		*n*=3		*n*=3		*n*=3		*n*=9		*n*=6	
**Time of dose administration**		9:00 h	24:00 h	9:00 h	24:00 h	9:00 h	24:00 h	9:00 h	24:00 h	9:00 h	24:00 h
**Capecitabine dose (mg/m²)**		375	625	475	800	600	1000	750	1250	750	1250
		Mean (%CV)	Mean (%CV)	Mean (%CV)	Mean (%CV)	Mean (%CV)	Mean (%CV)	Mean (%CV)	Mean (%CV)	Mean (%CV)	Mean (%CV)
**CAP**	$AUC_{0\text{-}5h}$ (µg*h/mL)	1.41 (13.5)	2.49 (25.3)	2.8 (31.8)	3.26 (29.4)	3.82 (18.6)	7.1 (7.3)	5.59 (70.3)	7.5 (66.4)	5.59 (33.1)	9.51 (39.2)
	C_{max} (µg/mL)	1.98 (22.7)	3.69 (68)	2.95 (54.6)	3.24 (55.2)	2.57 (34.6)	5.06 (10.7)	6.05 (77.2)	6.68 (53.3)	5.39 (62.3)	8.49 (54.1)
	t_{max} (h)	0.83 (34.9)	1.33 (108.3)	1.5 (88)	1.33 (43.6)	1.17 (65)	1.5 (33.3)	0.78 (56.4)	1.39 (50.4)	1.33 (77.4)	1.33 (39.1)
**dFCR**	$AUC_{0\text{-}5h}$ (µg*h/mL)	4.26 (10.3)	6.5 (38.3)	5.79 (35.6)	8 (26.5)	8.01 (13.6)	12.12 (11.3)	9.88 (22.4)	14.7 (36.8)	12.3 (14.8)	20.4 (39.7)
	C_{max} (µg/mL)	3.41 (26.4)	5.76 (85.1)	4.11 (62)	5.22 (54.6)	4.06 (36.9)	5.6 (18.4)	6.13 (29)	8.98 (46)	6.28 (35.8)	9.64 (51.1)
	t_{max} (h)	0.83 (34.9)	1.5 (88)	1.5 (88)	1.33 (43.6)	2 (50)	2 (0)	1.06 (36.8)	1.61 (37.3)	1.33 (69.9)	1.5 (30)
**dFUR**	$AUC_{0\text{-}5h}$ (µg*h/mL)	4.11 (12.2)	7.2 (27.1)	3.67 (19.9)	5.84 (27.2)	5 (29.8)	9.66 (23.5)	8.02 (23.1)	13.2 (43.5)	9.45 (28.9)	18.0 (42.8)
	C_{max} (µg/mL)	3.66 (20.8)	7.15 (70.3)	2.65 (45.3)	4.01 (59.6)	3.09 (41.1)	5.75 (16.3)	5.25 (44.2)	8.85 (61.2)	5.8 (59.5)	8.99 (57.2)
	t_{max} (h)	0.83 (34.9)	1.5 (88)	1.67 (68.9)	1.33 (43.6)	2 (50)	1.83 (15.8)	1 (43)	1.83 (41)	1.33 (69.9)	2 (42)
**5-FU**	$AUC_{0\text{-}5h}$ (µg*h/mL)	0.58 (6.9)	1 (33)	0.52 (5.8)	0.79 (11.4)	0.76 (31.6)	1.63 (27)	0.7 (77.1)	1.22 (77.9)	0.74 (20.3)	1.94 (105.7)
	C_{max} (µg/mL)	0.54 (18.5)	1.03 (71.8)	0.38 (26.3)	0.51 (45.1)	0.45 (37.8)	1.04 (46.2)	0.5 (80)	0.88 (97.7)	0.47 (48.9)	0.93 (107.5)
	t_{max} (h)	0.83 (34.9)	1.5 (88)	1.67 (68.9)	1.33 (43.6)	2 (50)	1.83 (15.8)	1 (43)	2.06 (61.2)	1.33 (69.9)	2.58 (55.4)
**FBAL**	$AUC_{0\text{-}inf}$ (µg*h/mL)	29.1 (33.7)	36.2 (6.5)	20.7 (56.7)	32.3 (73.9)	33.7 (39.1)	41.2 (63.9)	41.2 (62.3)	45.9 (68.5)	59.3 (60.8)	74.8 (58.5)
	C_{max} (µg/mL)	4.06 (20.7)	4.34 (18.7)	3.25 (28)	3.92 (39)	4.58 (46.3)	5.07 (36.1)	5.56 (28.4)	6.66 (22.7)	5.99 (29)	7.31 (37.3)
	t_{max} (h)	2 (0)	2.83 (66.8)	2.83 (66.8)	3.33 (45.9)	2.5 (34.8)	3 (0)	2.44 (45.1)	3.22 (33.9)	2.17 (77.9)	3.17 (30.9)

Abbreviations: CV, coefficient of variation; $AUC_{0\text{-}5h}$, area under the plasma-time curve up to five hours; $AUC_{0\text{-}inf}$, area under the plasma-time curve extrapolated to infinity; C_{max}, maximum plasma concentration; t_{max}, time to reach maximum plasma concentration; *n*, number of subjects

Supplementary Figure 1. Thymidine phosphorylase (TP) activity in peripheral blood mononuclear cells **(A)** at screening (within 3 days prior to treatment) and pre-dose at treatment day 7, and **(B)** at screening and end-of-treatment (EOT)(n=24).

A

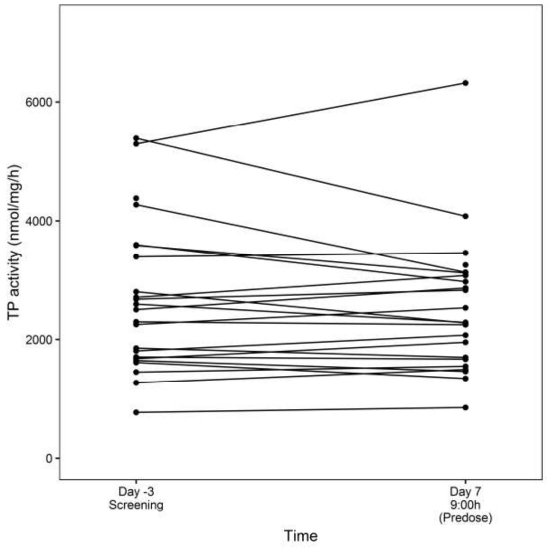

B

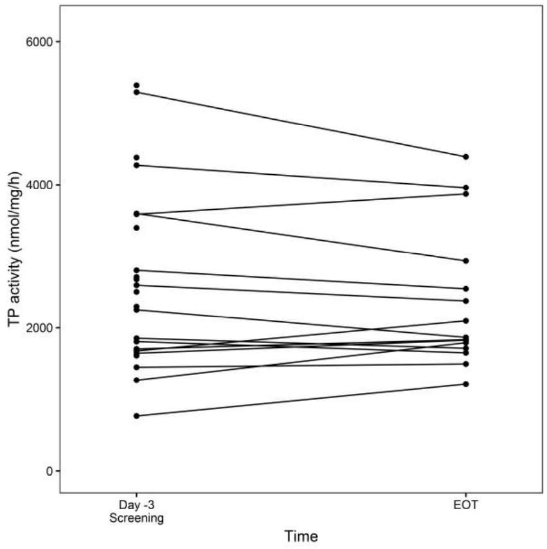

Supplement: Supplementary file 1 — (PDF 612 kb) [file 11095_2020_2828_MOESM1_ESM.pdf]
